# Persistent organic pollutants in Finnish reindeer (*Rangifer tarandus tarandus* L.) and moose (*Alces alces*)

**DOI:** 10.1186/1751-0147-54-S1-S11

**Published:** 2012-02-24

**Authors:** Anniina Suutari, Anja Hallikainen, Päivi Ruokojärvi, Hannu Kiviranta, Mauri Nieminen, Sauli Laaksonen

**Affiliations:** 1 University of Oulu, Department of Biology, P.O. Box 3000, 90014 Oulu, Finland; 2 Finnish Food Safety Authority Evira, Risk Assessment Research Unit, Mustialankatu 3, 00790, Helsinki, Finland; 3 National Institute for Health and Welfare, Department of Environmental Health, P.O. Box 95, 70701, Kuopio, Finland; 4 Finnish Game and Fisheries Research Institute, Reindeer Research Station, Toivoniementie 246, 99100 Kaamanen, Finland; 5 University of Helsinki, P. O. Box 33, 00014, Helsinki, Finland

## Abstract

**Summary:**

**Background**

The aim of this study was to determine 17 Polychlorinated Dibenzo-*p*-dioxin and Dibenzofuran (PCDD/F) and 12 Dioxin-like Polychlorinated Biphenyl (DL-PCB) concentrations in the tissues of Finnish terrestrial herbivore species, semi-domesticated reindeer (*Rangifer tarandus tarandus* L.), and wild moose (*Alces alces*), investigate transfer and accumulation of PCDD/Fs and DL-PCBs in milk of the lactating reindeer hinds, and explore contaminant concentrations in stillborn reindeer calves exposed via placental transfer to PCDD/Fs and DL-PCBs.

**Methods**

Reindeer and moose tissue sampling was focused in Finnish reindeer herding region. Reindeer milk samples were sampled in summer and autumn from reindeer hinds in experimental reindeer station in northern Finland. PCDD/Fs and DL-PCBs were analyzed using HRGC/HRMS method. The results are reported here as WHO-TEQ upper bound concentrations and congener-specific lower bound concentrations.

**Results**

WHO-PCDD/F- and PCB-TEQs in reindeer muscle and liver were generally higher in the calves than in adults. Concentrations in moose calves were lower than in reindeer calves, while in adult reindeer and moose the levels were equal. General PCDD/F congeners in reindeer muscle and liver were 23478-PeCDF, 123678-HxCDD and OCDD. In reindeer milk, the highest PCDD/F detected was OCDD, and it was common also in the moose muscle samples. A strong contribution of non-*ortho*-PCBs to WHO-TEQ was detected in all studied samples. The most dominating non-*ortho*-DL-PCB congener was PCB-126 in reindeer muscle, liver and milk. In moose muscle samples PCB-77 was the most abundant congener. Species-, individual- and tissue-specific accumulation of PCDD/Fs and DL-PCBs may be the result from varying extent and quality of exposure, and to some extent from different metabolic potential.

**Conclusions**

PCDD/Fs showed partly similar profiles in reindeer and moose muscle, reindeer liver and milk samples - indicating equal mode of bioaccumulation. A strong contribution of non-*ortho*-PCBs to WHO-TEQ was detected, although there were some differences in frequency of particular congeners in these species. Due to the harmonized sampling method the study offers the way to determine and compare the levels of PCDD/Fs and DL-PCBs in reindeer and moose tissues and examine the transfer and dynamics of dioxins and dioxin-like compounds in northern terrestrial food web.

## Background

Polychlorinated dibenzo-*p*-dioxins and dibenzofurans (PCDD/Fs) and dioxin-like polychlorinated biphenyls (DL-PCBs) are environmentally stable and toxic compounds, listed in Stockholm Convention [1] created to restrict and ultimately eliminate the production, use, release and storage of Persistent Organic Pollutants (POPs), and observed to exist globally in terrestrial and aquatic biota [2-4]. PCDD/Fs enter the environment solely as unintentional by-products from industrial and thermal processes, while DL-PCBs are merely intentionally produced chemicals that are released due to inappropriate disposal practices, accidents and leakages from industrial facilities [2,5].

As lipophilic substances, PCDD/Fs and PCBs absorb passively from the gastrointestinal tract, enter the circulation and distribute to high lipid tissues such as white adipose. Metabolism and excretion of these compounds are slow leading to accumulation of these substances in the organism. Metabolism or biotransformation can also create reactive intermediates that may cause tissue damage as a consequence of binding to proteins (e.g. transthyretin), lipids and nucleic acids [2,6,7]. PCDD/Fs and DL-PCBs are able to cause an array of adverse health effects, like cancer, damage to the central and peripheral nervous systems, reproductive and developmental disorders, and disruption of the immune and endocrine systems [8-11].

Tissue specific contamination and toxicokinetics are related to the physiology of the animals [12]. Differences in lipid distribution and lipid class profile, as well as lipid dynamics like fat accumulation and fasting, may affect tissue concentrations of POPs [4,13]. Weight loss in winter due to sub-maintenance feed intake is normal for e.g. free-living reindeer. A new equilibrium and redistribution of POPs in the body is established due to fasting and lipid loss and depending of the species, different tissues of animals can be targets for possible toxic effects [1]. In addition, milk production and lactation rely on fat depots, are significant route of elimination, and may therefore alter the distribution and decrease body burden of POPs in females [14].

Species-specific exposure, metabolism and accumulation of POPs result in different concentrations and contaminant profiles in the studied species. Ecophysiological factors, like feeding, may have an impact on the congener pattern seen in the animal body. E.g. PCDD/F profile in roe deer liver resembles that in conifer shoots (indicator for deposition via air), while profile in sheep liver is more similar with that of soil, indicating different eating behaviors [15]. Accumulation patterns may also vary among individuals of the same species, for example, Finnish reindeer fed only on natural pastures had higher PCDD/F- and DL-PCB concentrations than reindeer who had got supplementary feed [16]. Finnish reindeer and moose are interesting study objects because they share common living and contaminant deposition areas, and both are used as foodstuffs. Species-specific differences and elimination of dioxins in these economically, culturally and environmentally important *Cervid* species are highly interesting.

Age is considered to be one of the factors affecting POP levels. Generally, older individuals have higher POP levels than younger ones [17,18]. However, studies on reindeer in Finland have revealed higher concentrations in reindeer calf muscle than in adult reindeer muscle [19]. Accumulation of POPs in the early life stages of reindeer is supported by the observation of PCDD/Fs and DL-PCBs in Finnish stillborn reindeer calves [20]. Contaminant transfer from hind to fetus via placenta is occurring at critical and sensitive time period (body fat mobilization in mother and sensitive periods of organogenesis in fetus) resulting increased stress. Thyroid and steroid hormone systems are among the sensitive endocrine variables involved in the regulation of metabolic processes and development, and are potential targets of POPs both in hind and in fetus [21].

In addition to TEQs that are needed for risk assessment, the studies on individual congener profiles of PCDD/Fs and DL-PCBs in animal tissues give more detailed information on sources of exposure, species differences, individual variation, and differences among life stages and tissues. TEQ concentration, which is a measure of the total amount of PCDD/Fs and DL-PCBs adjusted for toxic potency, is a simplified method of assessing the risk of dioxin/PCB mixtures [22]. TEQ refers to the sum of the amounts of PCDD/Fs and DL-PCBs multiplied by their relative toxic potency as related to TCDD (the most toxic congener) according to the WHO [23].

The purpose of this study was to determine the concentrations and accumulation of 17 toxic PCDD/F congeners and 12 DL-PCB congeners in semi-domesticated reindeer and wild moose in order to identify the congener profiles of PCDD/Fs and DL-PCBs and reveal possible species- and tissue-specificity in accumulation. Standardized sampling method allowed a spatial survey of contaminant levels and profiles.

## Methods

**Figure 1:**
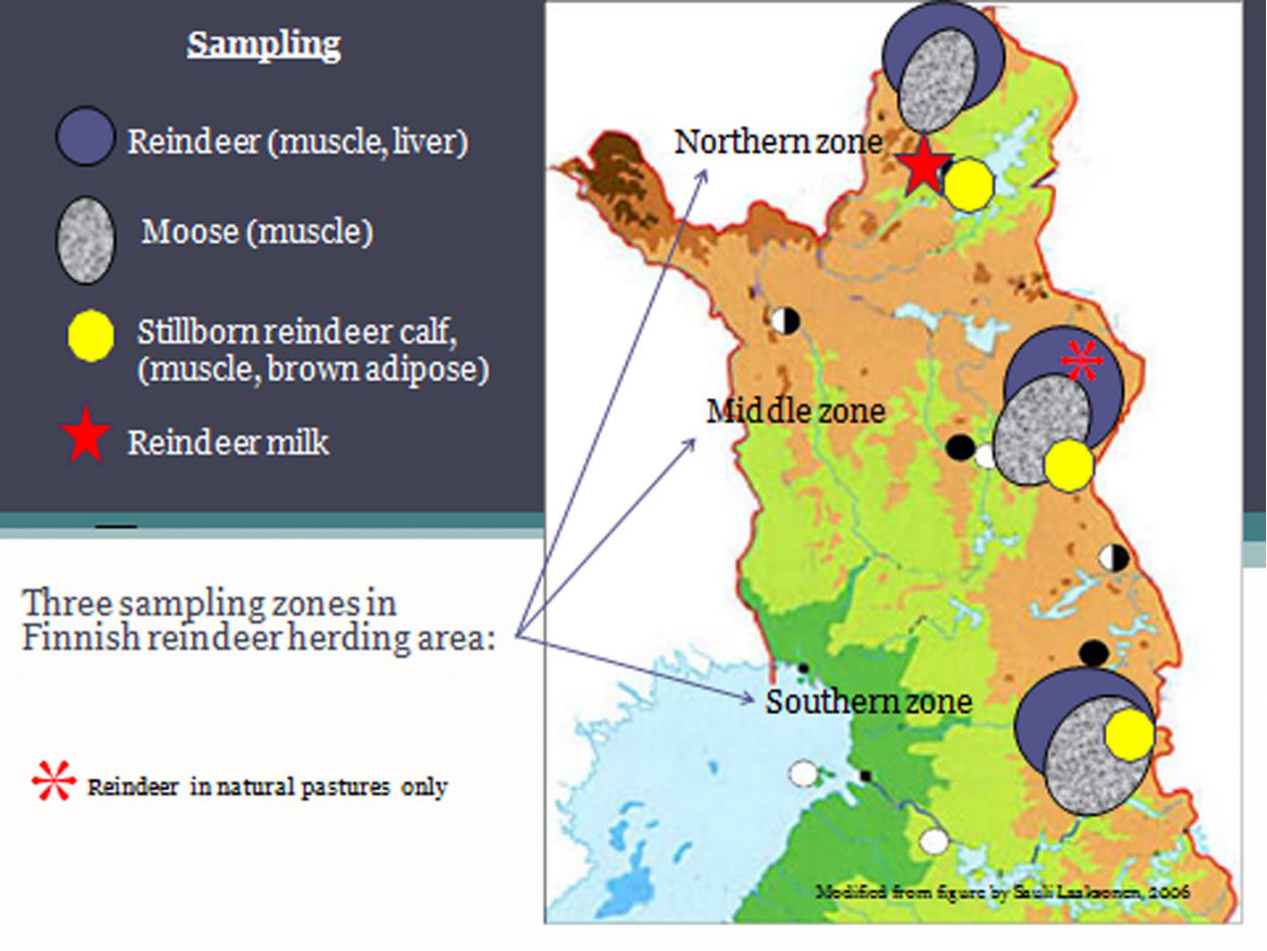
Sampling area.

The sampling area of reindeer and moose located in the sub-arctic northern Finland (Figure 1) and covered the reindeer herding region. The region was divided into three different sampling zones; the northern, the middle and the southern zone. The method of sampling was standardized hence allowing a comparison of the results between the different zones. The samples were built up in a ratio of carcass meat consumption. Concentrations of 17 toxic PCDD/F and 12 DL-PCB (dioxin-like PCBs; 4 non-*ortho* and 8 mono-*ortho* congeners) were measured from each sample (Table 1).

**Table 1:** Analyzed PCDD/Fs and DL-PCBs with toxic equivalent factors.

Congener	TCDD equivalent	Congener	TCDD equivalent
*PCDD/Fs*		*Non-ortho-PCBs*	
2378-TCDF	0.1	PCB-77	0.0001
2378-TCDD	1	PCB-81	0.0001
12378-PeCDF	0.05	PCB-126	0.1
23478-PeCDF	0.5	PCB-169	0.01
12378-PeCDD	1	*Mono-ortho-PCBs*	
123478-HxCDF	0.1	PCB-105	0.0001
123678-HxCDF	0.1	PCB-114	0.0005
234678-HxCDF	0.1	PCB-118	0.0001
123789-HxCDF	0.1	PCB-123	0.0001
123478-HxCDD	0.1	PCB-156	0.0005
123678-HxCDD	0.1	PCB-157	0.0005
123789-HxCDD	0.1	PCB-167	0.00001
1234678-HpCDF	0.01	PCB-189	0.0001
1234789-HpCDF	0.01		
1234678-HpCDD	0.01		
OCDF	0.0001		
OCDD	0.0001		

### Reindeer and moose muscle samples

The pooled muscle samples (weight 500 g) of reindeer (n=40) and moose (n=12) consisted of 200 g rump, 200 g rib and fore back, and 100 g shoulder muscle. The sampling was conducted using a clean knife and nitrile gloves to prevent contamination. The samples were stored in polyethylene bags in -20°C until analysis.

### Stillborn reindeer calves

The muscle samples (on average 270 g) of stillborn reindeer calves (n=11) were collected from spontaneously aborted calves. The samples consisted of rump, back and shoulder muscles. Brown adipose tissue (BAT) samples (n=3) were taken from stillborn calves of the middle and southern sampling zones. BAT samples were collected from the specific locations; around the shoulders, sternum, trachea and spinal cord, and from the abdominal and thoracic cavities. The samples weighted on average 20 g.

### Reindeer liver samples

The reindeer livers (n=14) were taken as solid tissues from reindeer calves and adult reindeer by using a clean knife and nitrile gloves to prevent contamination. The samples were stored in polyethylene bags in -20°C until analysis.

### Reindeer milk samples

The reindeer milk samples were gathered in the Kaamanen experimental reindeer station in Inari, localizing in the northern sampling zone. Milk samples were collected twice from the reindeer hinds (n=7), in the early summer and later in the autumn. The sample collection (30 ml in each) was performed by hand milking to pre-cleaned glass bottles. The milk collection was facilitated by using Oxytocin (10 IU, i.m.) to each hind. Milk samples were preserved frozen (-20°C) in dark until analysis.

### Chemical analysis

The analyses were performed at the accredited reference laboratory of chemistry at the National Institute for Health and Welfare in Finland. The requirements of standard EN ISO/IEC 17025 were completed. After homogenization the samples were freeze dried and fat was extracted with ethanol-toluene using Accelerated Solvent Extractor (ASE 300) equipment. The solvent was exchanged to hexane and the fat content was determined gravimetrically. The samples were defatted on an acidic silica column and purified and fractionated on alumina and carbon columns. PCDD/Fs and PCBs were analyzed with HRGC/HRMS using a selected ion monitoring mode (SIM) and resolution of 10 000. Further details of the analytical method can be found elsewhere [20].

### Reporting of the results

WHO-TEQ concentrations are reported as fat based upper bound concentrations (concentrations<LOQ=LOQ) to enable comparisons to the existing EU maximum level in the meat [24].The most abundant congener-specific PCDD/F and DL-PCB concentrations are reported as lipid based lower bound concentrations (concentration < LOQ =0). Blank samples covering the whole analytical procedure did not indicate any cross contamination.

### Statistical analysis

Statistical analysis was conducted using the SPSS 16.0 software. Analysis of variance (ANOVA) was used to detect significant differences among data set, when data were normally distributed. Kruskal-Wallis test was used if homogeneity of variances did not realize. The criterion for significance was *p* < 0.05.

## Results and discussion

### PCDD/Fs in reindeer and moose tissue samples

**Figure 2:**
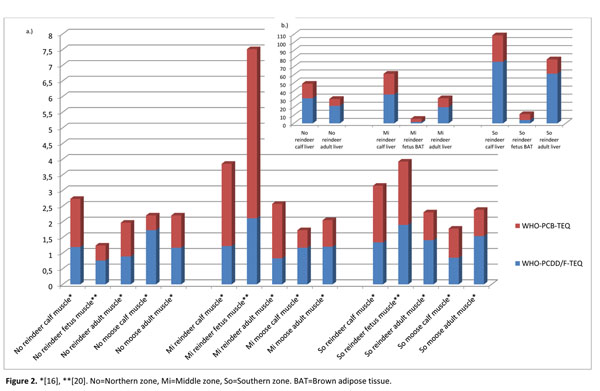
WHO-PCDD/F- and WHO-PCB-TEQs (pg/g fat) in a.) reindeer and moose muscle samples, and b.) in reindeer liver and brown adipose samples. *[16], **[20]. No=Northern zone, Mi=Middle zone, So=Southern zone. BAT=Brown adipose tissue.

**Figure 3:**
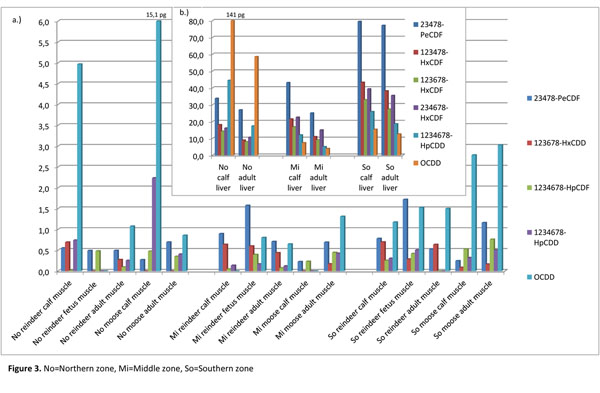
The most abundant PCDD/F congeners (pg/g fat) in a.) reindeer and moose muscle samples, and b.) in reindeer liver samples. No=Northern zone, Mi=Middle zone, So=Southern zone.

WHO-PCDD/F-TEQs in reindeer muscle (Figure 2 a) were quite equal (on average 1.2 pg/g fat) in the different sampling zones, being only moderately higher in the southern zone, and generally higher in the calves than in adults. WHO-PCDD/F-TEQs in reindeer liver (Figure 2 b) followed the same pattern than in muscle: higher levels in the calves and in the southern zone. The most prominent PCDD/F congeners in reindeer muscle samples were 23478-PeCDF, 123678-HxCDD and OCDD (Figure 3 a), of which 23478-PeCDF and OCDD were also characteristic for reindeer liver samples. In addition, 123478-HxCDF, 123678-HxCDF, 234678-HxCDF and 1234678-HpCDD were well representative in liver (Figure 3 b). For the comparison, some of these congeners found in reindeer liver, namely 23478-PeCDF, 1234678-HpCDD and OCDD, have been the most common congeners in roe deer liver samples in Germany [15]. Despite of the different zoogeographical origin of the species, roe deer’s feeding behavior resembles that of reindeer: variety of grasses, lichens, mushrooms, twigs and branches.

In this study, OCDD, with relatively low toxicity (TEF 0.0001) had a special property to exist at high concentrations (up to 140 pg/g fat) in reindeer muscle and liver in the northern area calf samples. This may indicate special and different exposure to OCDD from some wide scale emission source in the northern Finland, considering that adult reindeer in the same area also had proportionate high OCDD concentrations, especially in their liver. Also 2005 sampled reindeer calves in North-East Finland have shown high (160-fold) OCDD concentrations in muscle compared to many other existing PCDD/F congeners. The more toxic PCDD/F congener, 23478-PeCDF (TEF 0.5) concentrations were high in the calves and adult reindeer of the southern area, especially in their livers. This congener showed also high contribution in stillborn calves’ muscle tissue in the same area, and also in middle area’s stillborn calf muscle. Statistically significant differences (*P*<0.05) were seen in the concentrations of 2378-TCDF, 123478-HxCDD and 1234678-HpCDF, which showed to exist in higher levels in the calf, especially in stillborn calf, muscle samples in every sampling zone. On the contrary, 12378-PeCDF concentrations were significantly (*P*<0.05) higher in adult reindeer muscle samples. WHO-PCDD/F-TEQs in stillborn calves’ muscle were slightly higher in the middle and southern zone (on average 2 pg/g fat) than in the northern zone (0.8 pg/g fat).

Considering stillborn calves’ brown adipose tissue, it was seen that mean 23478-PeCDF concentration (2.4 pg/g fat) was overwhelmingly highest of any congeners (data not shown), and thus affected strongly (TEF=0.5) to the WHO-PCDD/F-TEQs, which were 1.7 and 4.1 pg/g fat in the studied middle and southern areas (Figure 2 b). However, also many other PCDD/F congeners existed in brown adipose and three of them (2378-TCDD, 123478-HxCDD and 123678-HxCDD) were lacking in muscle samples but represented in the brown adipose. On the other hand, 2378-TCDF, 1234678-HpCDD and OCDD seemed to exist in the muscle samples, but not in brown adipose, of stillborn reindeer calves.

Highly chlorinated congeners like 1234678-HpCDD and OCDD are generally considered to accumulate well in lipid-rich tissues, like brown adipose (fat content on average 30%), so the lack of these congeners is interesting. It may be the strong binding of these high chlorinated congeners to reindeer hind’s fat storages that restricts the transplacental diffusion to brown adipose of fetus. However, the muscle of fetus contained 1234678-HpCDD and OCDD in the middle and southern zones that may be indication of individual variations. There are indications of tissue-specific retention of PCDD/Fs in animals [25] that may be caused by structural specific binding sites and relative degree of absorption; those may partly explain different profiles in different areas and exposure conditions. 1234678-HpCDD and OCDD have observed to be the major PCDD congeners in the deposition samples in Pallas, in northern Finland [26].

WHO-PCDD/F-TEQs in moose muscle (Figure 2 a) (on average 1.3 pg/g fat) were equal with reindeer. The most dominating PCDD/F congeners in moose muscle tissue samples were OCDD, 23478-PeCDF, 1234678-HpCDF and 1234678-HpCDD (Figure 3 a). OCDD concentration (28 pg/g fat) was noticeable high in male calf sample from the northern zone. That was a parallel result to the reindeer calf muscle and liver samples from the same sampling zone, and indicated high OCDD exposure in the northern part of Finnish Lapland. Also 1234678-HpCDD showed an elevated concentration in northern zone’s moose male calf. A similar phenomenon was observed in reindeer calves from the northern zone.

Lower OCDD and 1234678-HpCDD concentrations in northern zone’s adult reindeer and adult moose may indicate different contaminant levels in emissions and exposure from food, in addition to individual physiological differences, but unlikely straight differences in metabolic activity and elimination potential. In middle and southern zones the exposure to especially OCDD may be lower and that is reflecting as the lower contamination both calves and adults. Concentration of 2378-TCDF was significantly (*P*<0.05) higher in the adult moose than in moose calves. That was an opposite result than with reindeer.

Very similar concentrations of 23478-PeCDF, 123478-HxCDF, 123678-HxCDF and 234678-HxCDF (TEF 0.1 in hexa-chlorinated congeners) in the livers of the southern zone’s reindeer calves and adult reindeer could indicate equal amount of binding sites for dioxins in the liver. On the other hand, considering the concentrations of the same congeners in the northern and middle zones, it is seen that calves had general higher levels in their livers than adult reindeer. However, the total concentrations were lower in the northern and middle zones than in the southern zone, even though the differences were not statistically significant.

### PCDD/Fs in reindeer milk samples

PCDD/Fs in reindeer milk samples (Table 2) showed generally similar profile than in muscle and liver samples. WHO-PCDD/F-TEQ showed a decreasing trend from the summer sampling (0.5 pg/g fat) to autumn sampling (0.4 pg/g fat) [20]. The most dominating congeners were 23478-PeCDF, 12378-PeCDD, 123478-HxCDF, 123678-HxCDD, 1234678-HpCDD and OCDD. There was a decreasing trend in reindeer milk samples from summer to autumn in most of the congener concentrations. Statistically significant (*P*<0.05) decrease were in the concentrations of 23478-PeCDF, 12378-PeCDD, 123478-HxCDF, 234678-HxCDF and 123678-HxCDD). However, 2378-TCDF, 1234678-HpCDD and OCDD showed significantly higher levels in the autumn than in the summer. In addition, OCDD, which showed the highest total contribution of PCDD/Fs, interestingly increased from zero in summer to on average 0.6 pg/g fat in autumn milk samples. Despite of increasing fat content of milk during the lactation period, the levels of some particular congeners were increased indicating no dilution effect.

**Table 2:** PCDD/Fs (pg/g fat), **non-*ortho*-DL-PCBs** (pg/g fat), and *mono-ortho-DL-PCBs* (ng/g fat) in reindeer milk samples.

Congener	Summer milk	Autumn milk
2378-TCDF	0.011	0.076
2378-TCDD	0.010	<LOQ
12378-PeCDF	0.033	<LOQ
23478-PeCDF	0.260	0.143
12378-PeCDD	0.143	0.016
123478-HxCDF	0.160	0.044
123678-HxCDF	0.063	0.024
234678-HxCDF	0.046	<LOQ
123789-HxCDF	<LOQ	<LOQ
123478-HxCDD	0.035	<LOQ
123678-HxCDD	0.124	0.023
123789-HxCDD	<LOQ	<LOQ
1234678-HpCDF	<LOQ	<LOQ
1234789-HpCDF	<LOQ	<LOQ
1234678-HpCDD	0.098	0.126
OCDF	<LOQ	<LOQ
OCDD	<LOQ	0.589
**PCB-77**	<LOQ	0.970
**PCB-81**	0.062	0.364
**PCB-126**	5.070	2.653
**PCB-169**	0.798	0.511
*PCB-105*	0.176	0.122
*PCB-114*	0.012	0.008
*PCB-118*	0.404	0.390
*PCB-123*	<LOQ	0.005
*PCB-156*	0.091	0.060
*PCB-157*	0.015	0.008
*PCB-167*	0.025	0.022
*PCB-189*	0.009	0.009

### DL-PCBs in reindeer and moose tissue samples

**Figure 4:**
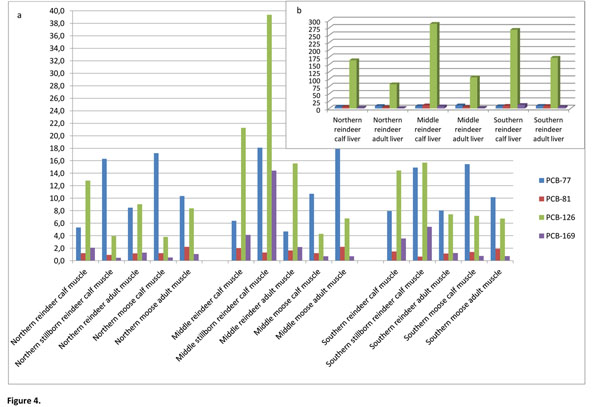
Non-*ortho*-DL-PCBs (pg/g fat) in a.) reindeer and moose muscle samples, and b.) in reindeer liver samples.

WHO-PCB-TEQs were higher in reindeer calf muscle (Figure 2 a) (on average 2.0 pg/g fat) than in adult reindeer (on average 1.2 pg/g fat). The highest level was seen in the middle sampling zone. The most dominating non-*ortho*-DL-PCB congener in reindeer muscle samples (Figure 4 a) was PCB-126, which highest concentration (39 pg/g fat) was detected in the middle zone’s stillborn calf. That individual contained also quite high concentration of PCB-77 (18 pg/g fat), which was the other very frequent congener in the studied population. PCB-77 was the only non-*ortho* congener being significantly (*P*<0.05) higher in stillborn calves’ muscle than in the other calves and adult reindeer. PCB-77 had also significantly (*P*<0.05) higher levels in stillborn calves’ muscle than in brown adipose tissue.

From non-*ortho* congeners, PCB-126 was the most dominating one in brown adipose (data not shown), followed by PCB-169. In addition, even if PCB-169 existed in many of the samples, it showed the highest level in the stillborn calf of the middle area (14.4 pg/g fat) (Figure 4 a). WHO-PCB-TEQ in stillborn calves’ brown adipose (on average 5.5 pg/g fat) was higher than in muscle (on average 2.6 pg/g fat). Overall, there was a strong contribution of dioxin-like non-*ortho*-PCBs (PCB-77, -81, -126 and -169) to total TEQ in the reindeer muscle samples. Of the mono-*ortho*-PCBs, PCB-118 was the most generally existing congener. In 38% of reindeer and moose tissue samples its concentration was above 1 ng/g fat (Table 3). The most congener containing mono-*ortho*-PCB profile was detected in middle area’s stillborn reindeer calf that indicates effective transfer and accumulation of these contaminants to fetus.

**Table 3:** Mono-*ortho*-DL-PCBs (ng/g fat) in reindeer and moose tissue samples.

Sample	PCB-105	PCB-114	PCB-118	PCB-123	PCB-156	PCB-157	PCB-167	PCB-189
Northern reindeer calf muscle	0.35	0.03	1.01	0.01	0.13	0.03	0.03	0.01
Northern reindeer calf liver	0.54	0.02	1.16	0.01	0.12	0.03	0.03	0.01
Northern stillborn reindeer calf muscle	0.13	0.01	0.36	<LOQ	<LOQ	0.01	<LOQ	<LOQ
Northern reindeer adult muscle	0.27	0.02	0.69	0.01	0.08	0.02	0.03	0.01
Northern reindeer adult liver	0.29	0.01	0.57	0.01	0.05	0.01	0.02	<LOQ
*Northern moose calf muscle*	0.13	0.01	0.42	0.02	0.03	0.01	0.02	<LOQ
*Northern moose adult muscle*	0.27	0.02	0.86	0.03	0.09	0.02	0.04	0.01
								
Middle reindeer calf muscle	0.65	0.05	1.72	0.02	0.28	0.07	0.06	0.02
Middle reindeer calf liver	1.11	0.04	2.22	0.02	0.28	0.07	0.07	0.02
Middle stillborn reindeer calf muscle	1.32	0.16	4.04	0.02	1.12	0.24	0.20	0.09
Middle reindeer adult muscle	0.43	0.03	0.96	0.01	0.14	0.04	0.05	0.01
Middle reindeer adult liver	0.67	0.02	1.03	0.01	0.12	0.04	0.04	0.01
*Middle moose calf muscle*	0.16	0.01	0.57	0.02	0.07	0.01	0.03	0.01
*Middle moose adult muscle*	0.25	0.02	0.79	0.04	0.07	0.01	0.04	0.01
								
Southern reindeer calf muscle	0.44	0.04	1.04	0.01	0.25	0.06	0.05	0.03
Southern reindeer calf liver	0.99	0.04	1.64	0.01	0.38	0.09	0.08	0.03
Southern stillborn reindeer calf muscle	0.33	0.05	0.71	0.01	0.41	0.08	0.05	0.05
Southern reindeer adult muscle	0.22	0.02	0.45	0.01	0.09	0.02	0.02	0.01
Southern reindeer adult liver	0.55	0.02	0.93	0.01	0.16	0.04	0.04	0.01
*Southern moose calf muscle*	0.32	0.02	0.94	0.04	0.10	0.02	0.04	0.01
*Southern moose adult muscle*	0.26	0.02	0.76	0.04	0.07	0.01	0.03	0.01

PCB-126 was clearly the most dominating DL-PCB congener in reindeer liver samples (Figure 4 b), followed by other non-*ortho*-PCB congeners PCB-77, -81 and -169, which concentrations were, however, much lower. The highest concentration of PCB-126, 400 pg/g fat, was exceeded in the middle zone’s female calf. TEF-value of PCB-126, 0.1, is the highest of DL-PCBs, thus influencing strongly to WHO-PCB-TEQ, which was higher in reindeer calf liver (on average 25 pg/g fat) than in adult reindeer liver (on average 12 pg/g fat) (Figure 2 b). The overall non-*ortho*-DL-PCB profile in reindeer liver fitted well to reindeer muscle samples. DL-PCB concentrations were generally higher in calf liver samples than in adult reindeer liver. That may indicate the calf liver functions being in state of effective accumulation of toxicants and weak detoxification resulting high concentrations of non-metabolized compounds in calf livers. However, concentrations of PCB-77 were significantly (*P*<0.05) higher in adult reindeer livers than in calves’ livers.

WHO-PCB-TEQ in moose calf muscle (Figure 2 a) was slightly lower (0.7 pg/g fat) than in adult moose (0.9 pg/g fat), that is opposite result than with reindeer. The most prominent non-*ortho* congener in moose muscle was PCB-77, followed by PCB-126 and PCB-81. Of mono-*ortho*- PCB congeners PCB-118 was the most detected one. There were no statistically significant differences between the DL-PCB concentrations in adult moose and moose calves. However, PCB-77 concentrations were significantly lower in female moose than in male moose in every zone, indicating excretion of compounds via lactation.

### DL-PCBs in reindeer milk samples

The most prominent non-*ortho*-DL-PCB congener in reindeer milk (Table 2) was PCB-126; this concerns both summer and autumn samples, when the mean concentrations were 5 and 2.7 pg/g fat, respectively. A clear decrease in the concentration of PCB-126 is seen from summer to autumn. Similar significant, (*P*<0.05) decreasing trend was seen also with non-*ortho*-PCB-169 (0.79 pg/g fat) in summer and 0.51 pg/g fat in autumn). However, PCB-81 increased significantly (*P*<0.05) from 0.06 pg/g fat in summer to 0.36 pg/g fat in autumn, and PCB-77 from zero in summer to 0.97 pg/g fat in autumn. This was due to one exceptional high PCB-77 concentration (6.79 pg/g fat) in autumn milk samples. PCB-118 remained steady (0.4 ng/g fat). Concentrations of PCB-114, -156 and -157 were decreased and PCB-123 increased significantly (*P*<0.05) from summer to autumn, concentrations being nevertheless very low.

The congener profile of DL-PCBs in reindeer milk was again fitted well to reindeer muscle and liver samples. Especially the most toxic DL-PCB-126 was well represented. WHO-PCB-TEQ in summer milk samples, on average 0.6 pg/g fat, decreased to 0.4 pg/g fat in autumn [20] that indicates transfer of DL-PCBs out of the body of female reindeer via lactational route. However, even emphasizing the importance of lactational transfer of persistent organic compounds it is worth of noticing that the highest concentrations of PCB-126 and -169 were found from stillborn calf that had got its body burden only via the placenta.

The mean fat content in autumn milk samples (26%) was significantly (*P*<0.05) higher than in summer milk samples (10%) that may has an influence to the lipid based concentrations detected. The fat content of reindeer milk normally varies from 11 to 30% during the lactation process [27].

## Conclusions

WHO-PCDD/F-TEQs were generally higher in reindeer calf muscle than in adult reindeer. PCDD/Fs showed partly similar profiles in reindeer and moose muscle, reindeer liver and milk samples-indicating equal mode of bioaccumulation. Among the most abundant congeners were 23478-PeCDF, 123478-HxCDF, 123678-HxCDF, 234678-HxCDF, 1234678-HpCDD and OCDD. However, stillborn calves‘ brown adipose was somewhat exception showing both accumulation and absence of congeners compared to muscle. In addition, more infrequent PCDD/F congeners existed in moose muscle samples; OCDD being the most visible. OCDD also showed interesting appearance to the reindeer milk PCDD/F set not until in the autumn. With DL-PCBs, a strong contribution of non-*ortho*-PCBs (PCB-77, -81, -126 and -169) to total TEQ was detected in all studied samples, although there were some differences in the frequent of particular congeners in the different species. Differences found in this study may indicate species-, individual- and tissue-specific accumulation of PCDD/Fs and DL-PCBs. In addition to metabolic potential, which concerns mainly liver, an extent and quality of exposure may explain the congener-specific accumulation.

## List of abbreviations used

HRGC/HRMS: High resolution gas chromatography/high resolution mass spectrometer; WHO-TEQ: Toxic equivalent defined by WHO; WHO-PCDD/F-TEQ: Toxic equivalent for 17 PCDD/Fs; WHO-PCB-TEQ: Toxic equivalent for 12 DL-PCBs; TEF-value: Toxic Equivalence Factor; LOQ: Limit of quantification.

## Competing interests

The authors declare that they have no competing interests.

## Authors’ contributions

Study design: SL. Data collection: SL, MN. Analysis of samples: PR. Data analysis: AS, SL. Statistical analysis: AS. Manuscript writing: AS. Critical review and approval of the final manuscript: AS, AH, PR, HK, MN, SL.
